# Workload effects of online consultation implementation from a Job-Characteristics Model perspective: a qualitative study

**DOI:** 10.3399/BJGPO.2022.0024

**Published:** 2023-02-22

**Authors:** Cordet Smart, Craig Newman, Lisa Hartill, Sian Bunce, John McCormick

**Affiliations:** 1 Department of Psychology, University of Exeter, Exeter, UK; 2 UXClinician Ltd, Exeter, UK; 3 Devon Sustainability and Transformation Partnership, Devon, UK; 4 Devon Clinical Commissioning Group, Devon, UK

**Keywords:** primary health care, general practice, technological innovations, remote consultation, workload

## Abstract

**Background:**

Online consultation (OC) was previously promoted by the NHS to solve primary care workload challenges. Its implementation was sped up during the COVID-19 pandemic. Workload effects are widely debated. Using a job design perspective may enhance understandings of workload effect.

**Aim:**

To qualitatively interrogate the workload experiences of primary care staff involved in OC implementation, using the Job Characteristics Model (JCM) to enable the following: a clearer understanding of the primary care staff psychological experiences; and recommendations informing the design of digital implementations and continued use.

**Design & setting:**

A qualitative interview study of GP practices using OC within South West England.

**Method:**

Thirteen participants representing seven practices completed JCM-based semi-structured telephone interviews. An abductive theoretically driven thematic analysis was completed.

**Results:**

Participants experienced different tasks pre- and post-implementation of OC, and adapted differently to them. Differences included the following: contact modality change, some administrative staff felt removed from patient contact; and in perceived autonomy, some GPs valued increased workload control. Variation in workload experience was affected by job role and practice context, and the form of and rationale for implementation. Use of a psychological model (the JCM) allowed clearer consideration of the effects of change, as well as OC on workload.

**Conclusion:**

Psychological theory may be helpful in interpreting workload effects of technology implementation such as OC. Designing change to include consideration of technology effects, psychological experiences, differences across roles, and individual and practice contexts may be important for technology implementation and evaluation of its workload effects.

## How this fits in

OC was believed to offer at least a part solution to rising workloads in GP practices. It has received mixed reactions from GP practices, with confusion over whether it has increased or reduced workload. Focusing on redesign of work when introducing digital technologies might have better effects in reducing workload and pressure. Using organisational psychology theory may help to predict where the difficult points might be and to improve staff wellbeing and reduce workload.

## Introduction

OC was previously promoted as a solution to high workloads and demand within UK primary care.^1^ OC encompasses multiple forms of digital consultation between a practice and a patient (webchat, online forms, text messaging, email, and video consultations).^2^ Its use increased over the past decade, then was mandated during the pandemic, with the impact on primary care workload being difficult to determine.^3^ Its continued adoption is, at the time of writing, potentially disrupted with government initiatives promoting a prioritisation of face-to-face consultation and a removal of the mandate. Nevertheless, primary care in the UK continues to struggle with demand, with major challenges surrounding staff wellbeing and retention, which is also an international issue.^4,5^

**Figure 1.**
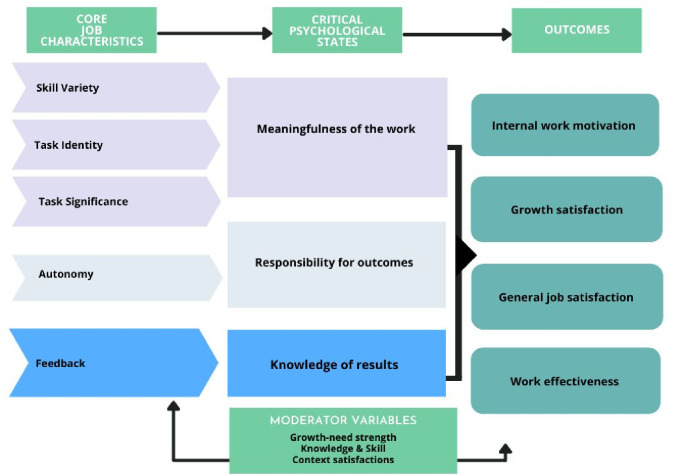
The Job Characteristics Model

Views of workload impact vary depending on staff role, the location of the GP practice, and perceptions of patient interest in the use of OC.^1^ The pandemic confounded effects with OC becoming a requirement to support social-distancing policies.^6^ As the pandemic subsides, there remain questions of how best to use OC within primary care and to consider its work impact.^7^ Internationally, there have been disparities in uptake and use, with higher adoption in both youngerand employed people before the pandemic,^8^ and increased use by older populations post-pandemic.^6^^,^^8^ Concerns from clinicians are repeatedly presented around maintaining clinical quality and workload demands.^8^ This article draws on the JCM^9^ (Figure 1) to better understand workload effects, focusing on its use within the UK.

### OC in the UK

A systematic search found pre-pandemic research into OC within the UK was limited, including 10 qualitative and mixed-method pre-pandemic evaluation studies of pilot implementations (scoping search; MEDLINE and CINAHL; terms: ‘online consultation’ and ‘primary care’) and one report^2^ (up to and including September 2020). Typically, articles reported patient usage rates across 6–15-month periods, one-off retrospective interviews with staff, and patient surveys. Implementation facilitators reportedly included a preference by younger, female patients. Barriers included increased workload fear^10–12^ and job role changes. Reported workload increases were major challenges for administration staff.^2^ No studies drew clear workload impact conclusions. Some staff were described as ‘protective’ over existing practice.^13^ Gradual change was recommended for staff and patients.^2^ Some staff showed less satisfaction than patients with OC, making staff an important research focus.^14^^,^^15^ Several studies focused on video consultation. Hammersley *et al*, for example, compared video and telephone with face-to-face consultations, concluding that telephone and video consults resulted in shorter, single-issue consultations.^16^ International studies, where digital services are charged to the patient, are difficult to compare with the UK; however, a US-based 2-year pilot study found that 40% of OCs resulted in no in-person visit.^17^

These studies included limited theory. Participant perspectives were reported at ‘face value’ with limited analysis of how experiences (for example, of organisational change) were relevant. This is except for Murphy *et al* who used normalisation process theory (NPT).^6^ NPT frames change as requiring the following series of actions: development of narrative coherence about the change; cognitive participation; a call to action; and reflexive component with staff for a complex intervention to operate. Having a theory enabled these authors to contextualise quotations relevant to participants’ positions in the change journey.^14^

Post-pandemic, as expected, OC studies increased (20 publications post-Sepember 2020 at time of writing). In 2020, Murphy *et al* (21 practices in Bristol and North Somerset) extended the use of NPT to interpret the data, enabling understanding that some experiences were related to the context of change.^6^ The study suggested OC was valuable during the pandemic, but some GPs considered it unsustainable in the longer term. Congruently, a quantitative meta-analysis of OC studies modelled OC as increasing workload by up to 30%.^3^ Some suggest staff in these studies may not have had time to adapt to change and that context (of the practice and change) is important for meaningful interpretation.^2^^,^^14^

### Using the JCM in primary care with OC

Understanding workload, like organisational change, may benefit from theory. Therefore, the JCM^9^ was used here.

The JCM proposes job design may consider skill variety (the perceived variety and complexity of skills and talents required to perform the job); task identity (the extent to which the job is seen as involving a whole, identifiable task); and significance (the extent to which the job affects the wellbeing of others), alongside autonomy, feedback, and meaning, which are important for positive work outcomes (for example, reduced turnover and increased job satisfaction).^18–21^ The extended JCM adds that motivation and contextual features, including values and social factors, are important.^22^^,^^23^ Applying the JCM^24^ to analyse workload may reveal aspects of staff job roles affected by organisational change (such as OC implementation). Analysing work differences before and post-change may allow better understanding of how to interpret perceptions of workload.^23^

The JCM has been used to examine primary care administrative roles, identifying a trade-off between increased cognitive load in task variety versus role clarity,^25^ and to enhance patient safety culture.^18,26^ Analysing the meaning of using OC in work may provide insight into how to redesign work; for example, some GPs raise clinical risk as a major concern for OC, while others experience OC as reducing the ‘messiness’ of GP consultations.^6^^,^^11^ Considering both factors in redesign of work might improve experiences (thus wellbeing and performance). Burrows *et al* highlighted that job design may not have been given enough consideration in primary care but may offer many benefits.^25^

The JCM further enables consideration of individual staff needs. It is sometimes combined with self-determination theory (SDT) to design work that enhances wellbeing,^27^^,^^28^ making relevant consideration of employee autonomy, relationships, and competence. Lack of autonomy has indeed been identified as a key factor in GP retention,^29^ suggesting these psychological components are relevant.

This study aimed to qualitatively interrogate workload experiences of primary care staff experiencing OC implementation, using the JCM to understand how work was perceived before, during, and post-implementation. The nuances of different staff roles and different implementation approaches were also considered.

## Method

### Design

A qualitative semi-structured interview study using abductive thematic analysis was conducted, with interviews and analysis focused on the JCM.^24^

### Participants

Recruitment was conducted during the COVID-19 pandemic between February 2021 and March 2021. Purposive sampling enabled representation of varied practice sizes, geographic areas, and Indices of Multiple Deprivation (IMD) levels, and involved multiple practitioner types from across a large area.^30^ All administrative and clinical staff involved in using OC were eligible to participate. Administrative staff, practice managers, and allied health professionals were a specific focus owing to their relative prior underrepresentation. Thirteen participants completed an online consent form to participate, with the link distributed by clinical commissioning groups (CCGs) and the research team to practices (Table 1).

**Table 1.** Details of practices and participants^a^

Practicepseudonym	Participantpseudonym	Area	List size, *n*	Role	Time in PC	Time in post	Training	Implementation model or stage
CitysideMedical	Nicola(PM-N)	Urban	≥20 000	Practice manager	11 years	11 years	Management experience	Hybrid,2 years
CompassMedical	Ben(GP-B)	Suburban+ urban	≥30 000	GP	Not disclosed	Not disclosed	GP training	Hybrid,3–4 years
Compass Medical	Anya(PM-A)	Suburban+ urban	≥30 000	Practice manager	9 years	9 years	Administration experience	Hybrid,3–4 years
Compass Medical	Natalie(AS-L)	Suburban+ urban	≥30 000	Admin team leader	16 years	16 years	Administration experience	Hybrid,3–4 years
GreenfieldsPractice	Richard(AHP-R)	Suburban+ rural	≥30 000	Urgent care practitioner	6 years	6 years	Paramedic	Total triage,2 years
GreenfieldsPractice	Jason(GP-J)	Suburban+ rural	≥40 000	GP	9 years	5 years	GP	Total triage,2 years
GreenfieldsPractice	Hannah(GP-H)	Suburban+ rural	≥40 000	GP	18 years	7 years	GP	Total triage,2 years
GreenfieldsPractice	Matilda(GP-M)	Suburban+ rural	≥40 000	GP	5 years	3 years	GP	Total triage,2 years
HilltopPractice	Megan(AHP-M)	Rural	≥14 000	Urgent care practitioner	18 months	18 months	Paramedic	Total triage,1 year
MeadowSurgery	Maria(PM-M)	Rural	12 000	Office manager	6 months	6 months	Managementexperience	Hybrid,≥6 months
MeadowSurgery	Lydia(AS-N)	Rural	12 000	Medical administrator	6 years	6 years	Administration experience	Hybrid,≥6 months
Moor AvenueSurgery	Mary(PM-M)	Suburban+ rural	10 500	Practice manager	34 years	34 years	Administration experience	Hybrid,2 years
SeaviewPractice	Paarvai(GP-P)	Rural	≥20 000	GP	10 years	3 years	GP	Hybrid,1 year

^a^All practices and participants are anonymised. PC = primary care.

### Interviews

Consent was confirmed at the beginning of the semi-structured telephone interview. Interviews were structured using the JCM, asking participants to describe the tasks, work, and relational components of their work, pre- and post-implementation of OC, and any perceived changes in meaningfulness and responsibility (Table 2).

**Table 2.** Semi-structured interview schedule

Interview questions	
1a	Firstly, thinking back to ‘before’ online consultation, can you describe the main tasks of your role?
1b	Can you describe the relational components of your role — that is, the ‘talking to people’, which is important but may not be captured by tasks? (For example, helping colleagues and talking to patients unrelated to their medical condition*)*
1c	Can you describe the different responsibilities of the role?
2a	Since the introduction of online consultation, can you describe any changes in the main tasks of your role?
2b	Can you describe any change to the relational components of your role — that is, the ‘talking to people’?
2c	Can you describe any changes to the different responsibilities of your role?
3	You have identified the following changes [*summarise*], are any of these issues linked to the change process of online consultation (setting it up or initiating it)?
4	Can you tell me which changes are likely to continue once online consultation is embedded? Do you see any of these changes as ongoing once online consultation is embedded?
5	Reflecting back, can you describe how the ‘meaning’ of your work may have changed, or not? Prompts: has online consultation changed how it feels your work affects others (team or patients), how work feels for you?
6	Can you tell me how you feel that your performance at work might have changed?
7	Are there other considerations you think are important for us to understand the impact of online consultation on workload?

### Analytic approach

An abductive thematic analysis was conducted^31,32^ using NVivo (version 12). A ‘bottom-up’ analysis began by identifying codes and grouping these into larger themes. There were four levels of coding reduction. This revealed themes that fitted the JCM, but also additional themes related to the meanings that participants experienced. After extensive coding by the first researcher, a second coder coded 20% of the data reaching 98% agreement. Differences were discussed and the JCM was then used to examine which themes fitted with the core job characteristics in a ‘top-down’ analysis. This thematic was theoretically driven as the source data were responses from a theoretically (JCM) designed interview.

### Credibility

Quality was assessed as assured through the following practices discussed below.^33^^,^^34^

#### Reflexivity

Weekly meeting of primary analysts discussing analysis and reflecting on personal reactions. Researchers discussed data sections where they felt drawn to empathise with participants, carefully reflecting on how to report these fairly.

#### Trustworthiness

An audit trail was maintained throughout.

#### Transferability

Diversity of practice demographics and OC implementation method was intended to capture challenges and insights relevant to multiple contexts.

#### Relevance

Consultation with the wider research team (a senior programme manager and a senior GP partner) enabled test of relevance.

## Results

**Figure 2.**
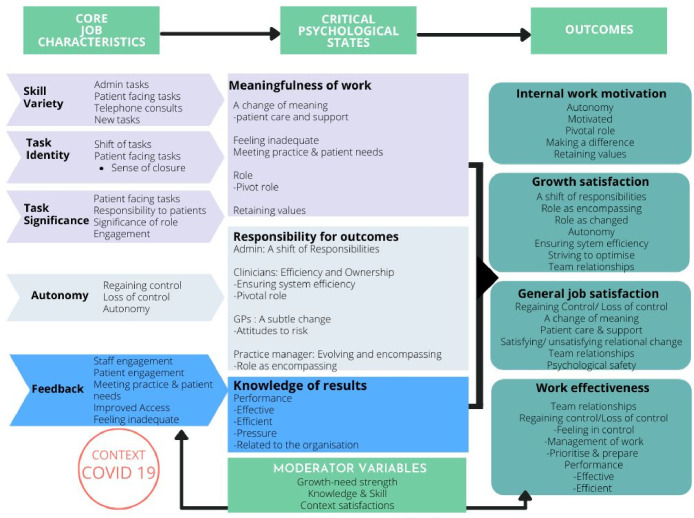
Findings mapped to Job Characteristics Model

This study qualitatively interrogated the workload experiences of primary care staff involved in the implementation of OC, examining perceptions of change in job task, relationships, and meaning, before, during, and post-implementation of OC. The main findings are mapped onto the JCM in Figure 2. There were multiple experiences expressed by participants, and it was clear that meanings of work changed differently for people in different roles. For example, many GPs‘ autonomy and control were important to their work, with perception of workload control increasing in a total triage system. Administrative staff, however, experienced a shift towards experiencing less meaningful relationships (reduced face-to-face appointments). Differences between practices in positive experiences were related to how OC was introduced, with dual-access approaches often accompanied by experienced loss of workload control. For practices already overwhelmed it was difficult to tease out effects of high demand, COVID-19, and OC implementation.

The main changes to each JCM component are discussed in turn, integrating role differences (see Supplementary Box S1) and practice differences (Table 3).

**Table 3.** Summary of differences in workload pressure experiences related to implementation model and stage

Implementation model or practice characteristics	Reduced workload pressure	Increased workload pressure
Total triage (*n* = 3): suburban (*n* = 1); rural (*n* = 1); and urban rural (*n* = 1)	Longer implementation period led to reduced pressure Refined triage reduced workload; for example, involving GPs, not just administration improved process Having the information presented before appointment improved GP experience of consultations It was possible to manage low-acuity appointments online, reducing the number of face-to-face appointments	Shorter implementation (6 months) Skill of taking OC over the phone or increased use of phone OC where patients were not proficient in the system It seemed to create more pressure for administrative staff than for GPs (but there are recruitment effects)
Hybrid model (*n* = 4): large urban (*n* = 1); suburban (*n* = 2); and rural (*n* = 1)	Change in workflow around travel immunisations and medication checks meant these did not need to be checked by a GP and reduced workload	Having OC and phoneline access as free-access routes (not triaged, not mandating triage using OC in detail, rather a more traditional approach) Putting OC in the right place — GPs would not get the right consult, increasing pressure on administrative staff OC taking a disproportionate time of the day for administrative staff, as they do not have dedicated time for it Felt out of control as there seemed to be no limit — running 24 hours a day

OC = online consultation.

### Skill variety

Changes in skill variety were discussed by all staff, with administrators discussing how they were now taking on more telephone work and some feeling that they had to ask more ‘clinical’ questions, which was difficult. For some GPs, OC meant a greater amount of variety in how people were accessing services and greater flexibility in responding to patients, with a trade-off around potential overload in switching tasks (see Supplementary Box S1).

### Task identity

For administrators, this was particularly challenging, as now much of the work was ‘*unseen*’ (AS-L) as people were directed to online resources, and decisions were made more via the technology. For GPs, the ways that the task was now conducted for some created ‘*unsatisfying relational change*’ (GP-P).

### Task significance

Task significance was different across roles. The two allied health professionals interviewed discussed role changes resulting in less *‘important work’* (AHP-M) such as triaging, now done by the *‘system’* (AHP-M). Two administrators described task significance as increasing as they now asked clinical questions over the telephone. Three GPs (GP-H, GP-M, and GP-J) included a description of ‘*some older GPs*’ as having concerns that the work was changing away from their role of consulting as a personal friend of the family:

*‘... a lot of my colleagues particularly I would say those older than me erm have really struggled with it because their whole way of consulting they’ve developed over a long time in the context of mostly seeing people face to face.‘* (GP-H)

### Workload perception

Workload was described in different ways. For example, in terms of pressure, one administrative team leader felt her team’s OC role took *‘the pressure off’* (AS- N) front-desk administrative staff.

Four clinicians measured their workload in terms of increased numbers of ‘non-significant’ referrals. Others (*n* = 8) described work as ‘different’.

All the participating GPs (*n* = 5) had previously experienced a series of much more intense face-to-face consultations, which OC reduced. For some, managing risk when using OC was initially challenging, with some colleagues being reported as still uncomfortable with this task.

It was indicated by some clinicians that OC enabled more comprehensive interactions with the patient, seen as both beneficial and satisfying:

*‘... with* [OC] *if you can get a decent if you can get the exact history of what’s going on then I think it’s really good if you can, when you reply back, you can put all these extra links in, certainly for things on phone calls which I may not always remember to do you can certainly add in lots of information.’* (AHP-R)

OC also increased opportunities for multidisciplinary working for some practice teams:

*‘... we’re certainly erm working more as a multidisciplinary team erm so communicating more with each other … because you get the online consult through and you have an opportunity to appraise the problem*.*‘* (GP-P)

### Autonomy

Autonomy was a major theme, particularly for GPs when discussing changes. People were stuck with a dilemma of experiencing feeling overloaded, particularly those from practices where the approach was an additional access route, compared with others who felt a greater amount of control over the workload (assisted by earlier triage) and able to *’decide when to do the* [OCs]’ (GP-M):

*‘I can do the difficult things earlier on in the day before I’m completely exhausted and can no longer think straight.‘* (GP-H)

Urgent care practitioners described greater role independence:

*‘I think there’s more* [independence]*, I can deal with a lot more jobs all the way through.‘* (AHP-M)

### Feedback

Feedback was not directly asked about at interview, but participants did describe OC effects. Participants described patients as having really engaged with OC during lockdown:

*‘We were using it before Covid hit and it it’s taken off more over the past 12 months.‘* (PM-N)

Staff engagement increased when staff witnessed OC being useful:

*‘... there was a little bit of you know, “why are we doing this type of thing“, which was inevitable with any change that you put in place — those things have been resolved as you started getting results.’* (PM-M)

Some were uncertain of their own level of sustainability owing to increased pressure attributed to OC:

*‘… I am still as motivated but … I’m not as motivated, ‘cos it’s really hard to find a resolution if I can’t have extra GP time, not willing to change how we do* [OC] *it is quite a difficult position to be in erm where you’re limited as what you can do*.*‘* (PM-A)

### Meaningfulness

The meaningfulness of work also appeared changed, with a change in relationships with patients being central to this. For example, for administrative staff who had less face-to-face contact with patients and began to feel more distanced:

*‘... that was the expression that was going around, basically we felt like we were working for a call centre.’* (AS-L)

*‘... doing it that way you’ve lost the erm the personal touch*.*‘* (AS-N)

GPs felt that meaning tied to patient relationships remained the same but maintaining this may require work:

*‘... for me the meaning would be around relationships with patients and I’m hoping that hasn’t changed really, that it’s just working out how to keep the important bits of that.‘* (GP-H)

### Job performance

In some practices, where OC was implemented to manage local escalating demand, performance in terms of the practice prioritising and supporting patients was enhanced, as many people received an electronic response:

*‘I think that we’re able to work much more efficiently because for a lot of those things I would be sending back some you know advice and guidance or emailing the pa — you know the patient.‘* (GP-J)

However, staff felt less connection with patients, some describing it as harder to support ongoing needs for chronic patients

*‘I do a lot of chronic pain work where patients have terrible pain, if they put pain as seven out of ten it says call an ambulance*.*’* (GP-B)

## Discussion

### Summary

This study examined OC implementation in primary care and experienced workload effects using the JCM. Applying JCM enabled a nuanced analysis of the experienced changes. Role, practice context, and individual differences affected how workload was experienced. Significant changes were described in tasks performed and their perceived significance. This did not seem to be something that staff were prepared for. This affected the meaning of people‘s work, particularly around relating to patients through technology above face-to-face contact. Role differences were significant for GPs and administrative staff; for example, administrative staff experienced their new role as removed, whereas GPs were more concerned with clinical accuracy. Practice context was relevant; for example, where a full triage model was implemented staff in this study perceived less overload following implementation than those creating dual-access routes.

### Strengths and limitations

The data should be treated as a small qualitative study with transferrable findings, not assuming generalisation. Recruitment was challenged by timescales and high pressure caused by the COVID-19 pandemic. However, it was strengthened by the use of a well-researched model where links between job characteristics and performance have been previously well established,^35^^,^^36^ therefore enhancing the theoretical interpretation of the work.

Analysis was subject to the research team, which included three psychologists, one project manager, and one senior GP and CCG member. Each had worked with OC in different professional ways. The main analysis was conducted by experienced qualitative and organisational psychologists. Data were discussed with the whole team in reflective groups, also examining researcher effects. Future research may benefit from a large quantitative study to examine job role changes post-technology introduction.

The JCM was used for brevity and parsimony,^9^ despite being limited in that it was originally developed with blue-collar workers. The current study focused on the original model as a framework to keep the interviews to a manageable length for busy GP practice staff. It might also be important to consider outputs in light of the extended version developed by Humphrey *et al*.^36^ A recent study critiqued the JCM’s level of consideration of context and engagement,^23^ and this might be critically examined in future work. There are some challenges around the evidence for perceived meaning of autonomy and meaningfulness as mediators,^37^ and some have suggested that work engagement might be a better mediator between job characteristics and performance.^38^ However, the current study suggests that considering the meanings and ‘sense making’ of individuals may be important. Critiques of autonomy have been that in manual workers increasing autonomy has led to reduced performance, but in some white-collar roles might lead to higher performance. The current study suggests that autonomy was given importance by GPs but not by other staff roles, and, for people in administative roles, there was greater meaning placed on the opportunity to chat to patients, informally, to improve relationships. This study therefore also contributes to the JCM literature in suggesting that qualitative studies of meaning may identify more complex relationships that need to be modelled in an enhanced JCM. They raise the question of what meaningful work is, rather than treating meaningful work as a singular psychological state that people experience. Extending this focus on ‘quantity’ of experienced meaningfulness into its content may provide valuable insights for leaders. Through understanding what makes work meaningful for staff they might better motivate staff to change and/or promote their engagement in change processes such as the introduction of OC.

### Comparison with existing literature

These findings reflect prior studies that OC implementation can lead to perceived workload increase, particularly for administration staff, and questioning of longer-term sustainability.^3,6,25^ However, the current study adds to knowledge by identifying qualitative differences in work. Arguably, quantifications of difference may not be helpful to account for the full experience of change nor COVID-19 impact.

Application of the JCM may suggest that understanding workload outcomes benefits from a qualitative appraisal of work changes, not just a quantitative evaluation. The qualitative change that staff experienced required staff to adapt their practice and get used to new ways of working, likely taking up additional cognitive resources for staff who may already be under pressure. Cognitive resource theory suggests that increased cognitive resource can be a precursor to burnout for individuals.^39^ Consideration of this risk when implementing OC and ongoing effects might be important at a design level. Similar difficulties have been identified for nurses post-implementation of digital medical records, where the cognitive burden is initially high.^39^ Therefore, identifying whether workload effects are a consequence of the technology or its implementation approach is challenging.

This JCM analysis adds to the NPT approach used by Murphy *et al* to understanding OC in the context of change.^6^ The NPT model focused on engagement of staff with change, through narratives, cognitive participation, and doing action. It treats change as something needing leadership and staff engagement. Multiple literature supports the need for staff acceptance of technology and engagement for success in healthcare change.^40^^,^^41^ This includes the importance of staff viewing the technology as ‘useful’ for the work being done. This JCM analysis suggests that in addition to perceptions of technology, the felt changes to daily work are important. This may have implications for development of ‘narratives of change’ to extend them beyond supporting the value of a technology, to encompass and prepare staff for experienced changes and redesign in the light of the technology. For example, to prepare staff for using triage in a different manner, as some of the present staff did including GPs in the process, which improved the overall work experience. Thus, when designing a change implementation, it may be important to plan for very specific changes in job roles.

Organisational experts repeatedly report change as complex, occurring across the organisation at multiple levels.^42^ Some liken it to a jazz concert, where improvisation is necessary.^43^ These areas of improvisation or ‘chaos’ might also be linked to the redefinition of new jobs and roles, which, in turn, could create challenges for those within organisations who may not be ready for change. They can challenge the relationship of individuals with organisations, who may perceive their psychological contract of how to work in an organisation has been broken, which can lead to disengagement.^43^ An organisational development approach, with discussion opportunities around role changes and to recognise the state of flux, might help people enter into ‘new’ roles brought on by changes such as OC. Such a reflective organisational approach may be more relevant for staff undergoing continuous change, such as in primary care, which in the UK has been in over-demand since at least 2009.^44^ The addition of the COVID-19 pandemic has heightened this demand and change, which may create a further need for job redesign.

The significance of changes in the tasks and roles of GP staff in the context of OC can also be understood better in terms of the issues of technology change more broadly. Sandkuhl *et al* ^45^ proposed that technological change needs an integrated model at a higher level, fitting the NPT approach and other recommendations in the OC literature.^6^ Additionally, they suggest that considering different forms of digital change is important for work design, particularly whether technology brings new processes and work roles, whether it uses those currently available, or whether there is a merge of both.^45^ In OC, this may be seen through different forms of implementation such as total triage, or as an additional access role. However, even with total triage, the system replaces the prior. Arguably, digital transformation can benefit from a total ‘review’ and may even be a rebuilding of primary care, which was reflected in a quote from a consultation session run by the researchers within the Devon Digital Accelerator programme in the UK (designed to rapidly implement OC): *‘A completely new way of dealing with medicine*.’

OC workload is important to understand for the sustainability of primary care and retention of staff.^44^^,^^46^^,^^47^ The demands on primary care staff are huge.^44^^,^^46^^,^^47^ Factors known to reduce burnout include supportive team relationships,^25^ autonomy,^48^ and meaningfulness and significance of work.^49^ Within caring roles, the meaning and altruistic side of work can be more important for motivation and protection against burnout than financial gain;^50^ therefore, changes to meaning, such as experienced reduction in face-to-face work and perceiving this as not enabling people to be helped, may reduce motivation. Further, there were variations in experiences of teamwork and support. Some practice managers and senior colleagues reported more challenges with engaging in team supportive activities, less face-to-face contact with colleagues, and less working physically in the same space, which made it harder for staff to feel supported by others in the team. Others found that OC enabled more time to discuss and ‘bounce off’ ideas about patients as there was time planned for managing workload. Future implementation of digital technologies might include considering enhanced staff support to mitigate effects of job demands on burnout.^51^^,^^52^

### Implications for practice

The JCM might help practices to consider job design for staff. The analysis higlighted effects of roles and work meaning on job performance and wellbeing. Reflecting on changes collaboratively may raise awareness and reduce the potential for staff to experience a breach in their psychological contract with work. In designing change, practices should consider the following: unique practice context; new possibilities the technology brings (new procedure versus transfer of approaches designed within different contexts [face-to-face not online]); and change in job design for staff. Change design (and maintenance) should focus on enhancing opportunities for autonomy (for example, use of OC to prioritise workload), identifying and avoiding opportunities for being ‘out of control’. The team need to acknowledge OC implementation as a ‘system’ change to manage staff expectations. Careful consideration should be given to opportunities for creating supportive and accessible teamwork experiences.

In conclusion, workloads in UK primary care are reportedly greater than ever, with the role of OC in this being uncertain. After its rapid implementation, perspectives on the value of OC vary hugely. This study suggests that ‘metrics’ of workload assessment (for example, patient calls) may overlook the qualitative differences in workload where OC is introduced. Using the JCM provided a guide for identifying differences in roles pre- and post-implementation. Differences were affected by job role (for example, GP and administrator) and practice context. Those implementing OC, or working with it, might consider not only the process of implementation and subsequent procedures, but also overarching system design. Collaborative discussion and job redesign may help to maintain engagement and improve workload experiences.
